# Supplementary material for: HIVprotI: an integrated web based platform for prediction and design of HIV proteins inhibitors
Source: J Cheminform. 2018 Mar 9;10:12. doi: 10.1186/s13321-018-0266-y (PMC5845081; doi:10.1186/s13321-018-0266-y)
Supplement: Supplementary file 2 — Additional file 2. Source code of HIVProtI web server. [file 13321_2018_266_MOESM2_ESM.zip › HIVprotI_Source-code/hivprotI/jsmol/jsvtest.htm]

Jmol/JSpecView Test 2 - full Object implementation


|  |  |  |
| --- | --- | --- |
| Get Data Types |  |  |
| Click on an H or C atom or select an IR mode. Once a spectrum is showing, you can click on selected regions as well, and there is a context menu (right-click) as well there. For 2D spectra, click on the spectrum, then use CTRL + to increase the contrast. | |  |
|  | | |
